# *spook* haploinsufficiency exacerbates aging-related motor impairments

**DOI:** 10.17912/micropub.biology.001871

**Published:** 2025-10-15

**Authors:** Veronica C. Ciliberto, Maya Solis, Carolyne Chepkosgei, Paul Rafael Sabandal, Kyung-An Han

**Affiliations:** 1 Biological Sciences, The University of Texas at El Paso, El Paso, Texas, United States

## Abstract

The global rise in aging populations heightens the risk of motor decline, reducing quality of life and increasing morbidities, yet aging-related motor impairments (AMI) remain poorly understood. Many steroid hormones synthesized by cytochrome P450 enzymes decline with aging but their role in AMI is unclear. Here we show that *spook* heterozygous flies with reduced brain ecdysone exhibit accelerated, aging-related declines in climbing performance in a stimulus-induced negative geotaxis assay, without affecting basal activity. These findings implicate ecdysone signaling as a critical regulator of aging-sensitive motor function and suggest a conserved steroid pathway contributing to AMI across species.

**Figure 1. spook deficiency exacerbates the aging-related decline in stimulus-induced negative geotaxis:**
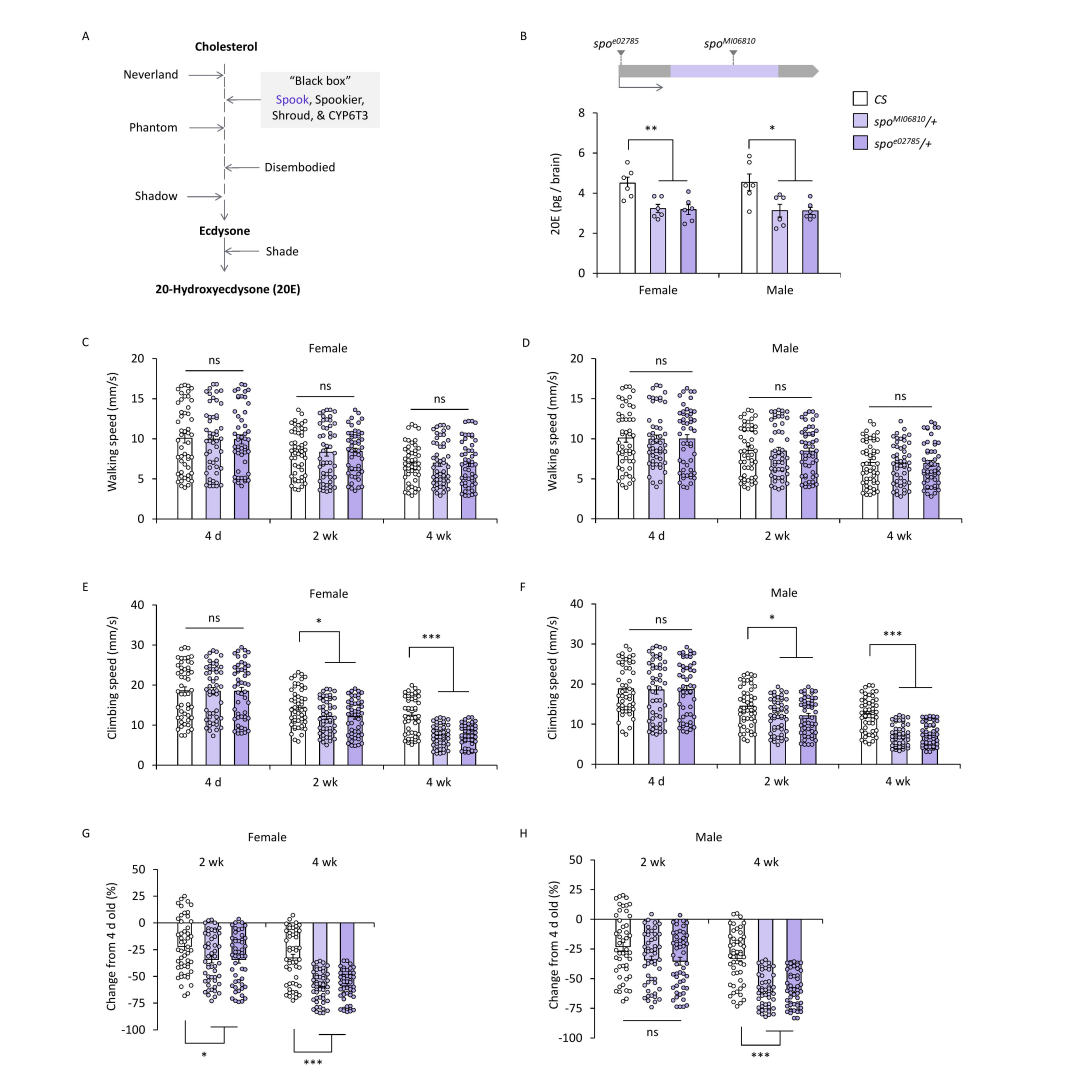
**(A)** The ecdysone biosynthetic pathway. Cholesterol is first converted to 7-dehydrocholesterol and then processed through a series of uncharacterized reactions, referred to as the “Black Box”, involving Spook and other enzymes. Subsequent hydroxylation steps are catalyzed sequentially by Phantom, Disembodied, and Shadow to generate ecdysone. The final conversion to the active hormone, 20-hydroxyecdysone (20E), is mediated by Shade. **(B)** Top: Schematic showing the locations of transgene insertions in the *spo* locus: *spo ^MI06810^* (MiMIC insertion in the coding region) and *spo ^e02785^* (piggyBac insertion in the noncoding 5’ UTR). The coding region is depicted by a purple box and noncoding regions in gray boxes (not to scale). The gray arrow indicates the direction of transcription. Bottom: 20E content in the brains of female and male *CS, spo ^MI06810^* /+, and *spo ^e02785^* /+ flies at 4 days old. Both heterozygous *spo* mutants had decreased brain ecdysone compared to *CS.* ANOVA with *post hoc* Dunnett’s test using *CS* as control: *, *p* < 0.05; **, *p* < 0.005; *n* = 6. **(C-D)** Average walking speed. **(C)** Female and **(D)** male *CS, spo ^MI06810^* /+, and *spo ^e02785^* /+ flies were assessed at 4 days (4 d), 2 weeks (2 wk), and 4 weeks (4 wk) of age. Walking speeds of *spo ^MI06810^* /+ and *spo ^e02785^* /+ flies were comparable to *CS* at all ages in both sexes. ANOVA with *post hoc* Dunnett’s test using *CS* as control. ns (not significant), *p* > 0.05, *n* = 50. **(E-F)** Average climbing speed. **(E)** Female and **(F)** male *CS, spo ^MI06810^* /+, and *spo ^e02785^* /+ flies were assessed at 4 days (4 d), 2 weeks (2 wk), and 4 weeks (4 wk) of age. *spo ^MI06810^* /+ and *spo ^e02785^* /+ flies exhibited slower climbing speeds compared to *CS* at 2 and 4 weeks in both sexes. ANOVA with *post hoc* Dunnett’s test using *CS* as control: ns, *p* > 0.05; *, *p* < 0.05; ***, *p* < 0.0001, *n* = 50. **(G-H)** Percent change in climbing speed relative to 4-day-old flies. **(G)** Female *spo ^MI06810^* /+ and *spo ^e02785^* /+ flies show greater decreases in climbing speed at 2 and 4 weeks compared to *CS* flies. **(H)** Male *spo ^MI06810^* /+ and *spo ^e02785^* /+ flies show greater decreases in climbing speed at 4 weeks compared to *CS* , but no significant difference at 2 weeks. ANOVA with *post hoc* Dunnett’s test using *CS* as a control: ns, *p* > 0.05; *, *p* < 0.05; ***, *p* < 0.0001, *n* = 50.

## Description

The global population aged 60 years and older is increasing at an accelerated pace (WHO, 2024), driving a rise in aging-associated health issues, including cognitive and motor decline and chronic disease (Aigbogun et al., 2017; Smith et al., 1999; Kauranen and Vanharanta, 1996; Samson et al., 2000; Stahl et al., 2021; Scarmeas et al., 2005). While cognitive decline has been extensively studied, aging-related motor impairments (AMI) remain poorly understood (Dewolf et al,. 2021; CDC, 2024; Kauranen and Vanharanta, 1996).

Multiple steroid hormones, including dehydroepiandrosterone (DHEA), androsterone, and sex hormones show aging-associated declines (Fiacco et al., 2019; Deltourbe et al., 2025; Walther et al., 2016). Steroid hormones, synthesized from cholesterol and metabolized by cytochrome P450 (CYP) enzymes (Payne and Hales, 2004), regulate diverse processes such as inflammatory response, sleep regulation, and cognition (Deltourbe et al., 2025; Terán-Pérez et al., 2012; Berman et al., 1997). Several CYP genes have been linked to cognitive dysfunction and dementia (Ghosh et al., 2016; Ferguson and Tyndale, 2011; Chace et al., 2012; Djelti et al., 2015; Nho et al., 2024), yet their role in AMI remains unknown.

In *Drosophila* , the major steroid hormone ecdysone, active as 20-hydroxyecdysone (20E), regulates development, reproduction, sleep and memory like mammalian steroid hormones (Caldwell et al., 2005; Carney and Bender, 2000; Schwedes et al., 2011; Ishimoto et al., 2009; Ishimoto and Kitamoto, 2010). However, its functions in aging and dementia are understudied. Since flies exhibit aging-related deficits in negative geotaxis, flight, and movement (Gargano et al., 2005; Rhodenizer et al., 2008; Miller et al., 2008; McKenzie-Smith et al., 2025; Soyam and Kannan, 2024), we investigated ecdysone’s contribution to AMI.

To address this, we examined flies carrying mutations in the *spook* ( *spo* ) gene. *spo* is a member of the *Drosophila* Halloween gene family and encodes a CYP enzyme required for ecdysone biosynthesis (Niwa et al., 2014; Namiki et al., 2005; Chavez et al., 2000) ( Figure 1A ). Two heterozygous *spo* mutant alleles *spo ^MI06810^ /+* and *spo ^e02785^ /+* containing a transgene insertion in the coding region and non-coding 5’ UTR, respectively showed significantly reduced levels of 20E in the brain compared to *Canton-S* ( *CS* ) controls ( Figure 1B : ANOVA; females, *p* < 0.01; males, *p* < 0.05; *n* = 6). Notably, ecdysone levels did not differ between female and male brains in *CS* and *spo/+* mutants ( Figure 1B : two-way ANOVA, *p* = 0.834; *n* = 6).

To assess basal activity, we measured exploratory walking speed in FlyTracker at three ages: 4 days (young), 2 weeks (mid-age), and 4 weeks (early-old). All genotypes showed aging-dependent decrease in walking speed (Figures 1C and 1D: ANOVA; *R* ^2^ = 0.135 for *CS* females, *R* ^2^ = 0.126 for *spo ^e02785^ /+* females; Kruskal-Wallis; *p* = 0.001 for *spo ^MI06810^ /+* females, *p* < 0.0001 for *CS* males, s *po ^MI06810^ /+* males, and s *po ^e02785^ /+* males; *n* = 50). At each age, *spo ^MI06810^ /+* and *spo ^e02785^ /+* flies exhibited walking speeds comparable to *CS* controls (Figures 1C and 1D: Kruskal-Wallis; *p* = 9.79 at 4 d; *p* = 0.975 at 2 wk; *p* = 0.955 at 4 wk; *n* = 50). No sexual dimorphism in walking speed was detected across genotypes or ages (Mann-Whitney or 2-sample *t* -test; *CS* : *p* = 0.942 at 4 d, *p* = 0.764 at 2 wk, *p* = 0.964 at 4 wk; *spo ^MI06810^ /+: p* = 0.964 at 4 d, *p* = 0.659 at 2 wk, *p* = 0.994 at 4 wk; *spo ^e02785^ /+: p* = 0.796 at 4 d, *p* = 0.964 at 2 wk, *p* = 0.997 at 4 wk; *n* = 50). Together, these results indicate that *spo* deficiency does not affect basal activity.

We next examined the stimulus-induced negative geotaxis (SING) in *spo/+* flies across the three age groups. Consistent with previous studies (Gargano et al., 2005; Rhodenizer et al., 2008), *CS* flies displayed an aging-dependent decline in climbing speed (Figures 1E and 1F: Kruskal-Wallis, *p* < 0.0001 for *CS* females; ANOVA, *p* < 0.0001 for *CS* males; *n* = 50). Like *CS* , *spo ^MI06810^ /+* and *spo ^e02785^ /+* flies also showed aging-dependent declines in climbing speed (Figures 1E and 1F: Kruskal-Wallis: *p* < 0.0001 for *spo ^MI06810^ /+* females and males, *p* < 0.0001 for *spo ^e02785^ /+* females and males; *n* = 50). At 4 days old, *spo ^MI06810^ /+* and *spo ^e02785^ /+* flies displayed climbing speeds comparable to *CS* controls (Figures 1E and 1F: Kruskal-Wallis; *p* = 0.989 for females, *p* = 0.971 for males; *n* = 50). However, at 2 weeks and 4 weeks old, they exhibited significantly greater declines in climbing speed compared to *CS* (Figures 1E and 1F: Kruskal-Wallis; *p* = 0.031 for 2 wk females, *p* < 0.0001 for 4 wk females, *p* = 0.043 for 2 wk males, *p* < 0.0001 for 4 wk males; *n* = 50). Although this pattern was observed in both sexes, no sexual dimorphism was detected (Mann-Whitney or 2-sample *t* -test, *CS* : *p* = 0.746 at 4 d, *p* = 0.993 at 2 wk, *p* = 0.940 at 4 wk; *spo ^MI06810^ /+: p* = 0.915 at 4 d, *p* = 0.992 at 2 wk, *p* = 0.705, at 4 wk; *spo ^e02785^ /+* : *p* = 0.871 at 4 d, *p* = 0.978 at 2 wk, *p* = 0.951 at 4 wk; *n* = 50).

To further quantify aging-related changes, we compared the decline in climbing speed from 4 days to 2 weeks old and from 4 days to 4 weeks old between *CS* and *spo/+* flies (Figures 1G and 1H). Female *spo ^MI06810^ /+* and *spo ^e02785^ /+* flies exhibited significantly greater declines in climbing speed at both intervals ( Figure 1G : Kruskal-Wallis; *p* = 0.038 for 2 wk females; ANOVA; *p* < 0.0001 for 4 wk females; *n* = 50). In males, no significant difference was detected between 4 days to 2 weeks old ( Figure 1H : Kruskal-Wallis; *p* = 0.061; *n* = 50), but *spo/+* males showed a significantly greater decline in climbing speed by 4 weeks old ( Figure 1H : Kruskal-Wallis; *p* < 0.0001; *n* = 50). Despite this sex-specific patterns at 2 weeks old, no sexual dimorphism was detected in the magnitude of decline (Mann-Whitney or 2-sample *t* -test; *CS:* *p* = 0.833 at 2 wk, *p* = 0.920 at 4 wk; *spo ^MI06810^ /+* : *p* = 0.942 at 2 wk, *p* = 0.642 at 4 wk; *spo ^e02785^ /+* : *p* = 0.893 at 2 wk, *p* = 0.791 at 4 wk; *n* = 50). Together, these results indicate that *spo* deficiency exacerbates the aging-dependent decline in climbing capacity and suggest that ecdysone is essential for maintaining SING performance.

Overall, this study reveals the role of *spo,* and likely ecdysone signaling, in AMI, specifically SING deficits. SING is a measure of locomotor activity, in which flies climb upward in response to sudden, intense stimulus. This behavior is regulated by specific dopaminergic neuron clusters that innervate the mushroom body, a key cognitive center in the fly brain (Sun et al., 2018). Since *spo* deficiency impaired SING performance but not basal activity, we hypothesize that ecdysone contributes to higher-order brain functions that control motor behavior. Ecdysone is the principal steroid hormone in flies, whereas humans produce more than 20 steroid hormones across development and aging (Vazakidou et al., 2024). Although it remains unclear whether deficits in specific steroid hormones contribute to AMI in humans, our findings provide evidence for a potential role of steroid hormones in AMI.

Motor deficits are not confined to aging. AMI can be exacerbated by neurodegenerative diseases such as Alzheimer’s disease (AD), which affects more than 57 million people globally (Andrade-Guerrero et al., 2024; Sayyid et al., 2024; WHO, 2025). While motor impairments are typically most evident in the intermediate and late stages of AD, early manifestations have been used to predict disease development and severity in older adults (Ogawa et al., 2018; Simpkins et al., 2024; Burns et al., 2010; Scarmeas et al., 2005). Individuals with AD also exhibit altered levels of steroid hormones and changes in the expression of neurosteroid biosynthetic enzymes (Djelti et al., 2015; Weill-Engerer et al., 2002; Luchetti et al., 2011). Moreover, genetic variations in CYP genes have been associated with heightened AD risk and earlier onset of disease (Djelti et al., 2015; Chace et al., 2012). The mechanisms of AD-related motor impairments remain unknown; however, our findings implicate steroid hormones and warrant further investigation.

## Methods

**Fly strains and maintenance**

The wild-type *Canton-S* ( *CS* ) was used as controls. *spook* ( *spo* ) mutant alleles, *spo ^MI06810^* (BDSC #42182) and *spo ^e02785^* (BDSC #18081), were obtained from Bloomington Drosophila stock center (BDSC; Bloomington, IN). Flies were maintained on standard cornmeal/sucrose/agar/yeast medium. Newly eclosed flies were collected under CO _2_ within two days and housed in mixed-sex groups. For aging experiments, flies were transferred to fresh food every 2–3 days until 4 days (young), 2 weeks (mid-age) and 4 weeks (early-old age). Two days prior to behavioral assays, flies were separated by sex, transferred to fresh vials, and kept at 25° C with 50% humidity under a 12 h light/12 h dark cycle.

**Ecdysone analysis**

To measure 20-Hydroxyecdysone (20E), the active form of ecdysone, in fly brains, we used a commercially available 20E Enzyme Immunoassay kit (A05120; Bertin Pharma, Montigny-le-Bretonneux, France). Brains were dissected in ice-cold hemolymph-like HL3 solution (70 mM NaCl, 5 mM KCl, 20 mM MgCl2, 10 mM NaHCO3,5mM trehalose, 115 mM sucrose, and 5 mM HEPES, pH 7.2) (Sabandal et al., 2020). One hundred brains were pooled in 50 µl of EIA buffer (Bertin Pharma, Montigny-le-Bretonneux, France) and then homogenized with a Kontes micro tissue grinder (Thermo Fisher Scientific, Waltham, MA). Homogenates were transferred to fresh tubes, centrifuged at 14,000 rpm for 10 min at 4 °C, and stored at -80 °C until assay. Ecdysone quantification was conducted according to the manufacturer instructions (Bertin Pharma, Montigny-le-Bretonneux, France). Multiple independent biological replicates (e.g., six sets) derived from independent crosses were analyzed.

**FlyTracker experiments**

To measure exploratory walking behavior, individual flies were placed in a clear rectangular chamber (60 mm L X 60 mm W X 15 mm H), allowed to acclimate for 1 min, and then recorded for 5 min (Sabandal et al., 2025). Videos were analyzed using the Viewer3 tracking software (BiObserve Technologies, Bonn, Germany), which enables tracking and measurement of average walking speed (mm/s). The average walking speed was calculated from the first minute of recording after acclimation.

**Stimulus-induced negative geotaxis (SING) assay**

To assess climbing capacity, individual flies were placed in vertical tubes and allowed to acclimate for 1 min. After acclimation, each tube was tapped downward five times in rapid succession and climbing behavior was recorded and analyzed using Viewer3 (BiObserve Technologies, Bonn, Germany). Average climbing speed was calculated based on the time required for each fly to reach the top of the tube.

**Statistical Analysis**

Statistical analyses for ELISA, exploratory walking, and SING assays were performed using Minitab software (Minitab, State College, PA). Parametric data were analyzed using either a two-tailed *t* -test or ANOVA with *post hoc* Dunnett’s multiple comparison test. Non-parametric data were analyzed using the Kruskal-Wallis test followed by Mann-Whitney tests. *p* > 0.05 was considered not significant (ns) while *p* < 0.05, *p* < 0.005, and *p* < 0.0001 were denoted by *, **, and ***, respectively.

## Reagents

**Fly strains**

**Table:** 

***Drosophila* Strain **	**Genotype**	**Source /Identifier**
*Canton-S*	*Canton-S*	Han Lab
* spo ^MI06810^*	y[1] w[*]; Mispo[MI06810]/TM3, Sb[1] Ser[1]	BDSC #42182
* spo ^e02785^*	w[1118]; PBacspo[e02785]	BDSC #18081

**Materials**

**Table:** 

**Reagents/Resources**	**Source /Identifier**
Ecdysone (20-E) ELISA kit	Bertin Pharma (Cat#A05120)
Viewer3	BiObserve Technologies, Bonn, Germany http://www.biobserve.com/behavioralresearch/
Minitab	Minitab, State College, PA https://www.minitab.com/en-us/
